# GC-MS Metabolite and Transcriptome Analyses Reveal the Differences of Volatile Synthesis and Gene Expression Profiling between Two Apple Varieties

**DOI:** 10.3390/ijms23062939

**Published:** 2022-03-09

**Authors:** Shunbo Yang, Dongmei Li, Shanshan Li, Huijuan Yang, Zhengyang Zhao

**Affiliations:** 1 College of Horticulture, Northwest A & F University, Yangling 712100, China; yangsb@nwafu.edu.cn (S.Y.); lidongmei@nwafu.edu.cn (D.L.); lishanshan8090@nwafu.edu.cn (S.L.); 2 Shaanxi Engineering Research Center of Apple, Yangling 712100, China

**Keywords:** aroma, apple, GC-MS, transcriptome sequencing, gene expression

## Abstract

Aroma is a key quality attribute of apples, making major contributions to commercial value and consumer choice. However, the mechanism underlying molecular regulation of aroma formation genes and transcription factors remains poorly understood in apples. Here, we investigated the aroma volatile profiles of two apple varieties with distinctive flavors using headspace solid-phase microextraction (HS-SPME) combined with gas chromatography–mass spectrometry (GC-MS). A total of 35 volatile compounds were identified in Granny Smith and Jonagold apples. Aldehydes were the most abundant volatiles contributing to the aroma in Granny Smith apple while esters were the dominant volatile compounds in Jonagold apple. In order to know more about the expression levels of aroma-related genes involved in the metabolic pathways, transcriptome sequencing of these two different apple varieties was conducted utilizing the Illumina platform. In total, 94 differentially expressed genes (DEGs) were found in the fatty acid metabolism, amino acid metabolism, the mevalonate pathway and phenylpropanoid pathway. Furthermore, compared to the Granny Smith apple, the expression of multiple genes and transcription factors were upregulated in the Jonagold apple, which might play important roles in the synthesis of aroma volatile compounds. Our study contributes toward better understanding on the molecular mechanism of aroma synthesis in apples and provides a valuable reference for metabolic engineering and flavor improvement in the future.

## 1. Introduction

Apple (*Malus* × *domestica* Borkh.) is one of the most widely cultivated fruits in temperate regions of the world [1]. The characteristic flavor of apple fruits depends upon taste and aroma, while aroma is often considered the dominant component and greatly affects consumer acceptance [2,3,4]. The aroma profile in apple results from the interaction of many volatile compounds, including esters, alcohols, aldehydes, ketones, acids and terpenoids, with only a few that contribute significantly to the apple sensory quality [5,6,7]. The esters, particularly those with six to ten numbered carbon chains including combinations of acetic, butanoic, and hexanoic acids with ethyl, butyl, and hexyl alcohols, have been considered as the primary contributors to apple fruity aroma [8]. Alcohols are the second most important compounds in ripe apples after esters, the most abundant being 2-methyl-1-butanol, 1-butanol, 1-hexanol and 1-propanol [9]. Aldehydes are produced at relatively high amounts in immature apples, such as hexanal, trans-2-hexenal and butanal [10]. Nowadays, more than 300 volatile compounds have been measured in apple fruits [11]. All the volatiles are of great importance for the complete characteristic aroma profile of apples.

Volatile compounds in apples are mainly synthesized from fatty acids, amino acids and carbohydrates [9,12]. Fatty acids are major precursors of aroma volatile compounds in apples [5]. Lipoxygenase (LOX) and β-oxidation are the two key pathways involved in fatty acid metabolism for the formation of straight-chain aldehydes, alcohols and esters, whereas branched-chain aldehydes, alcohols and esters are mainly derived from amino acids isoleucine (Ile), leucine (Leu) and valine (Val) [13,14]. These amino acids are branched compounds of aliphatic nature and are synthesized in chloroplasts [15]. In addition, terpenoids come directly from carbohydrate metabolism and are synthesized from mevalonate (MVA) and methylerythritol phosphate (MEP) pathway, with α-farnesene as the most prominent terpenoid compound in apples [16]. Phenylpropenes such as estragole and eugenol, derived from the phenylpropanoid pathway, also have the influences on the apple flavor and aroma [17].

The composition and concentration of volatile compounds in apples are specific to the varieties [18,19]. ‘Granny Smith’ and ‘Jonagold’ are two apple varieties with distinctive flavors and aroma profiles as well as characteristic skin colors. ‘Granny Smith’ as a late maturing, green-skinned apple variety, is believed to have descended from French crab apples grown in Australia [20]. The volatile profile of Granny Smith apples is characterized by the producing low levels of ethyl and propyl esters and free alcohols such as 2-methylbutanol [21]. ‘Jonagold’, a cross between ‘Jonathan’ by ‘Golden Delicious’, has gained popularity in North America, Northern Europe and China for its high yield and dessert quality [22]. The volatile profile of Jonagold exhibits an abundance of hexyl acetate, hexyl butyrate, butyl hexanoate and α-farnesene [18]. Hence, Jonagold is considered one of the most aromatic apples while the Granny Smith is regarded to be one of the least aromatic apples [23,24]. These two varieties represent two phenotypic extremes in volatile compound production and emission by apples. Although previous studies have measured the aroma profiles in Jonagold, Granny Smith and some other apple varieties [18,22,24], little is known about the transcriptional regulation mechanisms of volatile compounds and the expression patterns of aroma-related genes in apples.

Identifying and investigating the molecular mechanism behind volatile synthesis is essential to improve aroma production through metabolic engineering. In the present study, we evaluated the volatile profiles in Granny Smith and Jonagold apples using HS–SPME coupled with GC–MS and established transcriptome datasets of two extreme aroma apple varieties to gain a deeper insight into the molecular mechanism of volatile compounds biosynthesis and emission. The objectives of this study were to analyze the profiles of aroma volatile compounds in Granny Smith and Jonagold apples, and investigate the regulatory mechanism underlying key gene expression patterns with the volatiles production in apples.

## 2. Results

### 2.1. Phenotype and Physiological Traits of Granny Smith and Jonagold Apples

**Figure 1:**
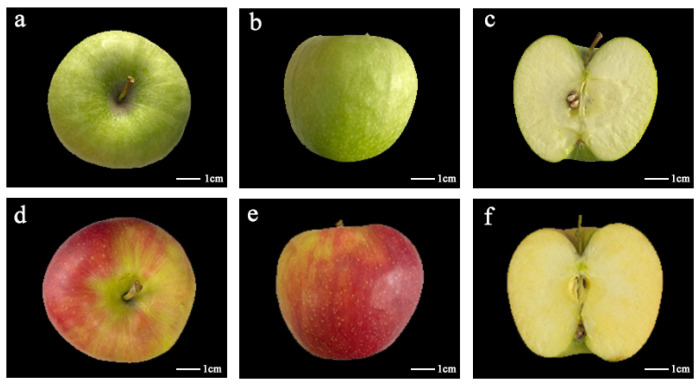
Phenotypes of Granny Smith and Jonagold apples. (**a**–**c**) Phenotype of the Granny Smith apple. (**d**–**f**) Phenotype of the Jonagold apple.

The phenotypes of Granny Smith and Jonagold apples were observed. As shown in Figure 1, the apple variety of Granny Smith was characterized with the green skin, in comparison with the red stripe skin in Jonagold. Color perception is the result of three parameters L* (lightness), a* (red–green), and b* (yellow–blue). The values of color parameters L*, a* and b* were 63.52, −17.62 and 37.95 in Granny Smith, while these values in Jonagold were 58.62, 13.38 and 29.13. Moreover, there are some other differences in physiological traits between these two apple varieties (Table 1). The fresh weight (FW) of Granny Smith and Jonagold apples was 205 and 240 g, respectively. The total soluble solid (TSS) of Granny Smith (13.7 °Brix) was lower than that of Jonagold (14.2 °Brix). On the contrary, the titratable acidity (TA) of Granny Smith (0.40%) was higher than that of Jonagold (0.32%). Firmness is another important factor to evaluate the quality of apple fruits [25]. The firmness of Granny Smith was 7.05 kg/cm^2^, higher than that in Jonagold (6.58 kg/cm^2^) (Table 1). Notably, the internal ethylene of the Granny Smith apple was 5 μL/L, lower than that of Jonagold (62 μL/L). However, the CO_2_ production rate in Granny Smith (70 μmol/kg·s) was higher than the 48 μmol/kg·s in Jonagold apple (Table 1).

### 2.2. Volatile Compound Profiles in Granny Smith and Jonagold Apples

**Figure 2:**
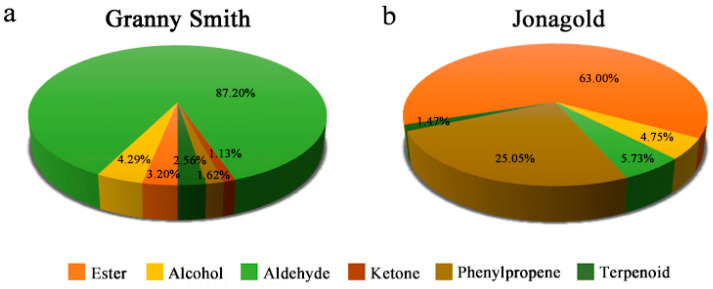
Percentage (%) of each type of volatile compounds in Granny Smith and Jonagold apples. (**a**) Volatile compounds in Granny Smith. (**b**) Volatile compounds in Jonagold.

Aroma is an essential sensorial property, which determined by the volatile compounds. In the present study, a total of 35 volatile compounds were identified in Granny Smith and Jonagold apples, including 20 esters, 4 alcohols, 8 aldehydes, 1 ketone, 1 phenylpropene and 1 terpenoid (Table 2). Aldehydes, occupying 87.20% of the total volatiles (56.14 µg/kg FW), were the most abundant type of volatile compounds contributing to the apple aroma in Granny Smith, following by alcohols (4.29% of the total volatiles; 2.76 µg/kg FW) and esters (3.20% of the total volatiles; 2.06 µg/kg FW) (Figure 2a; Table S2). By contrast, the main types of volatile compounds in Jonagold were esters (63.00% of the total volatiles; 1333.35 µg/kg FW), followed by phenylpropene (25.05% of the total volatiles; 530.16 µg/kg FW) and aldehydes (5.73% of the total volatiles; 121.27 µg/kg FW) (Figure 2b; Table S2). The apple variety of Granny Smith contained only 16 kinds of volatile compounds, while Jonagold had 31 kinds of volatiles (Table 2). Moreover, 12 volatile compounds were common to the two apple varieties. Among those volatiles, the contents of hexyl acetate, hexyl butanoate and hexyl 2-methylbutyrate in the Granny Smith apple were 1.25, 0.35 and 0.46 µg/kg FW, respectively, lower than that of 885.38, 27.19 and 31.95 µg/kg FW in the Jonagold apple. Furthermore, volatiles such as 1-hexanol, hexanal and (*E*)-2-hexenal showed lower contents in Granny Smith than that in Jonagold (Table 2). Additionally, compared to the Granny Smith, Jonagold had higher contents of estragole (530.16 µg/kg FW) and α-farnesene (31.15 µg/kg FW). In comparison with the volatiles detected in these two apple varieties, four volatile compounds were unique to the Granny Smith apple, including 2-hexyn-1-ol, (*Z*)-3-hexenal, (*Z*)-2-heptenal and 1-octen-3-one. There were 19 volatile compounds unique to the Jonagold apple, such as butyl acetate, 2-methylbutyl acetate, 1-butanol and (*E*)-2-decenal (Table 2).

### 2.3. RNA-seq, Assembly and DEGs Functional Annotation

To explore the molecular mechanisms underlying differences in aroma volatile compounds between Granny Smith and Jonagold apples, six cDNA libraries (three biological replicates) were constructed from these two apple varieties and sequenced utilizing the Illumina sequencing platform. Approximately 6.15–6.94 G of clean bases were produced from Granny Smith and Jonagold apples (Table S3). After data filtering and quality trimming, 41.01–46.29 million clean reads were obtained through sequencing of the cDNA libraries prepared from these two apple varieties. The Q30 percentage was over 93.01% for each sample and the average GC content was 47.03% for all six libraries (Figure S1; Table S3). Furthermore, average error rate of the sequenced bases was less than 0.03% (Figure S2). A total of 33.21–40.12 million reads were mapped to the apple reference genome, with match ratios in the range of 78.29–92.30% (Figure S3; Table S4).

**Figure 3:**
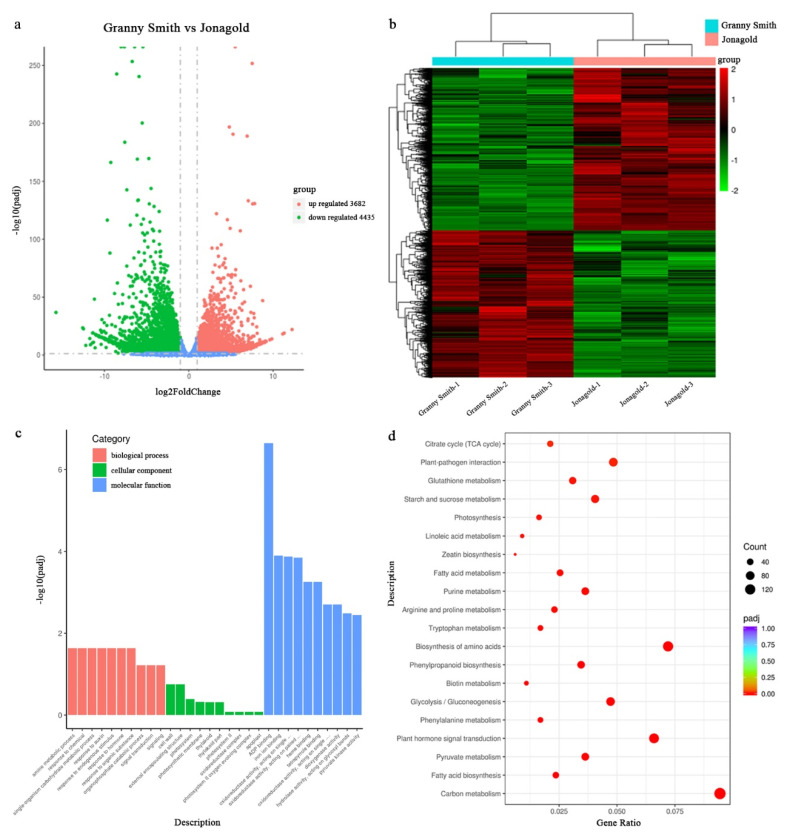
Analysis of differentially expressed genes (DEGs) between Granny Smith and Jonagold apples. (**a**) Volcano plot of DEGs. (**b**) Hierarchical cluster analysis of DEGs. (**c**) Gene ontology (GO) classifications of DEGs. (**d**) Kyoto Encyclopedia of Genes and Genomes (KEGG) pathway enrichment of DEGs.

FPKM methods were used to analyze gene expression patterns in Granny Smith and Jonagold apple libraries (Figure S4). Comparing these two types of libraries with respect to the FPKM calculation, 20,481 and 20,794 genes were identified in the cDNA libraries, while 1954 and 2267 genes were expressed specifically in Granny Smith and Jonagold apples, respectively (Figure S5). A total of 8117 DEGs were detected, including 3682 upregulated genes and 4435 downregulated genes (Figure 3a). To investigate the differences in gene expression patterns between Granny Smith and Jonagold, DEGs were subjected to hierarchical clustering analysis of transcript abundances using the FPKM values, and these DEGs had different transcriptome profiles in the two apple varieties (Figure 3b).

In order to know more about the biological function of the DEGs, Gene Ontology (GO) and Kyoto Encyclopedia of Genes and Genomes (KEGG) enrichment analyses were performed. According to the GO enrichment analysis, these DEGs were classified into three categories including biological process, cellular component and molecular function. The most abundant terms of each category were ADP binding, single-organism carbohydrate metabolic and cell wall process (Figure 3c). Based on the KEGG enrichment analysis, DEGs were matched and assigned to 122 pathways. The most enriched pathways were carbon metabolism, biosynthesis of amino acids, plant hormone signal transduction and fatty acid metabolism. Moreover, the pathways of phenylalanine metabolism and linoleic acid metabolism were also enriched (Figure 3d).

### 2.4. Expression Levels of Genes Involved in Fatty Acid and Isoleucine Metabolism Pathways

**Figure 4:**
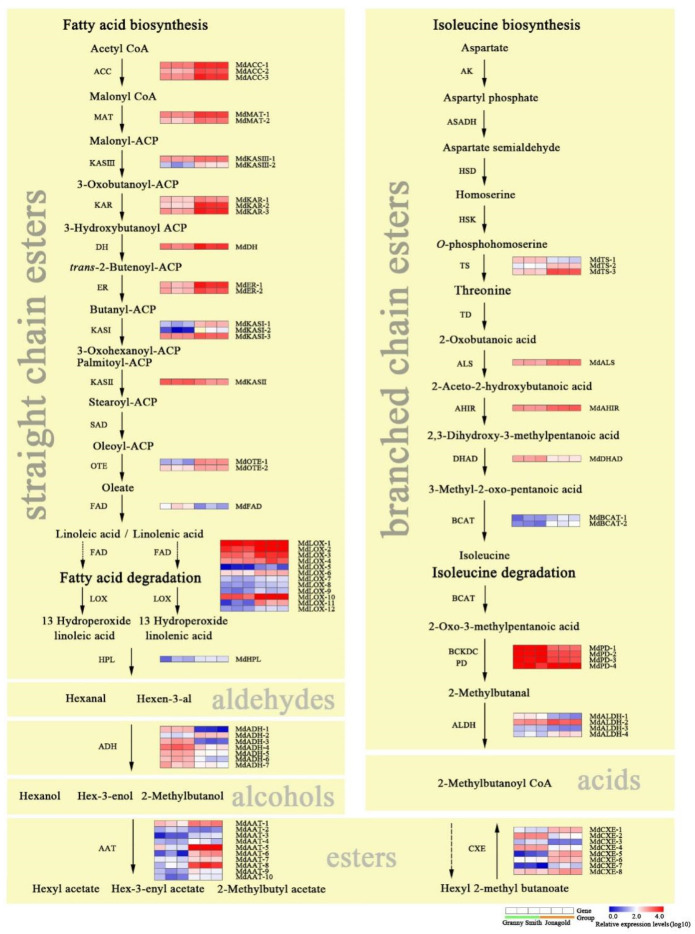
Expression profiles of differentially expressed genes (DEGs) involved in fatty acid and isoleucine metabolism pathways between the Granny Smith and Jonagold apples.

Fatty acid and isoleucine metabolism are the two main pathways for synthesis of aldehydes, alcohols and esters in apples [6]. In this study, we identified 50 DEGs related to fatty acid metabolism and 24 DEGs associated with isoleucine metabolism (Figure 4). Among them, most of the DEGs showed higher expression levels in the Jonagold apple than that in the Granny Smith apple. In the pathway of fatty acid biosynthesis, the expression levels of *ACC*, *MAT*, *KAS* and *FAD* genes exhibited higher expression levels in the Jonagold apple in comparison with in the Granny Smith apple. Similarly, the DEGs *KAR*, *DH*, *ER* and *OTE* showed the same expression patterns. Lipoxygenase (LOX) is the initial step of ester biosynthesis in fatty acid metabolization [26]. Twelve *LOX* DEGs were identified in our study, most of which were significantly upregulated in the Jonagold apple. Hydroperoxide lyase (HPL) is the last step toward aldehyde biosynthesis [27]. Compared to the Granny Smith apple, the expression levels of HPL gene showed higher in the Jonagold apple (Table S5). Alcohol dehydrogenases (ADH) can catalyze the aldehydes to the corresponding alcohols. Seven DEGs encoding ADH were detected in our dataset, most of which were significantly upregulated in the Granny Smith apple. Alcohol acyltransferase (AAT) is the key enzyme for the biosynthesis of esters [9]. Ten *AAT* DEGs exhibited higher expression levels in the Jonagold than that in the Granny Smith apple.

Straight-chain esters are synthesized from the fatty acid metabolism pathway while branched-chain esters are produced by the pathway of isoleucine metabolism [13]. Aldehyde dehydrogenase (ALDH) and carboxylesterase (CXE) are the two important enzymes for the synthesis of branched-chain esters. In our study, four DEGs encoding ALDH and eight DEGs encoding CXE were identified, most of which were upregulated in the Jonagold apple compared to the Granny Smith apple. In addition, some DEGs encoding acetolactic synthetase (ALS), acetohydroacid isomeroreductase (AHIR) and branched-chain aminotransferase (BCAT) also had enhanced mRNA levels in the Jonagold apple (Figure 4; Table S5).

### 2.5. Expression Levels of Genes Related to Sesquiterpene and Phenylpropanoid Metabolism

**Figure 5:**
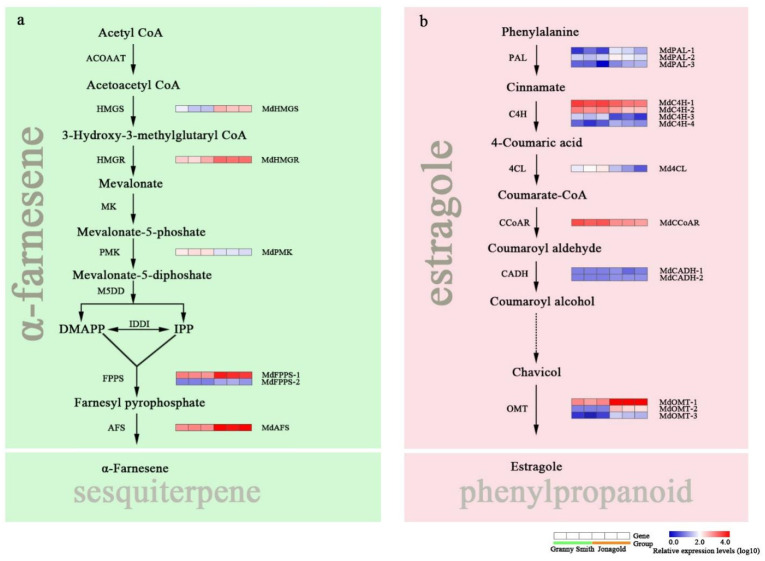
Expression profiles of differentially expressed genes (DEGs) involved in sesquiterpene and phenylpropanoid metabolism pathways between the Granny Smith and Jonagold apples. (**a**) Biosynthetic pathway for α-farnesene. (**b**) Biosynthetic pathway for estragole.

Sesquiterpenes are the most prominent terpenoids in apples [28]. In our study, α-farnesene was the most abundant sesquiterpene accumulated in Granny Smith and Jonagold apples. A total of nine enzymatic steps are involved in the biosynthetic pathway of sesquiterpene (Figure 5a). Compared to the Granny Smith apple, two DEGs encoding HMG-CoA synthase (HMGS) and HMG-CoA reductase (HMGR) exhibited higher expression levels in the Jonagold apple (Table S6). In cytosol, isopentenyl diphosphate (IPP) and its allylic isomer dimethylallyl diphosphate (DMAPP) catalyzed by the enzyme farnesyl pyrophosphate synthase (FPPS) results in the formation of farnesyl pyrophosphate. The *FPPS* genes were significantly downregulated in the Granny Smith apple in comparison with the Jonagold apple. Additionally, the final step in sesquiterpene biosynthesis in apple is catalyzed by α-farnesene synthase (AFS). In this study, one DEG was identified as the *AFS* gene and showed a higher expression level in the Jonagold apple than that in the Granny Smith apple (Figure 5a).

Estragole, which belongs to the phenylpropenes, is synthesized from the phenylpropanoid pathway in apples. In our study, a total of 14 DEGs were obtained in this pathway (Figure 5b; Table S6). At the beginning of this pathway, four DEGs encoding phenylalanine ammonia lyase (PAL) were upregulated in the Jonagold apple (Figure 5b). However, compared to the Granny Smith apple, the *C4H*, *4CL* and *CCoAR* genes showed lower expression levels in the Jonagold apple. The final step in estragole biosynthetic pathway involves a class of methyltransferase. Three DEGs encoding O-methyltransferase (OMT) were identified in the dataset and all of them exhibited higher expression levels in the Jonagold apple compared with the Granny Smith apple.

### 2.6. Expression Levels of Transcription Factors Associated with Volatile Compounds

Transcription factors (TFs) play many important roles in regulating gene expression in various plant biological processes including the production of volatile compounds. In this study, we found that 30 DEGs were putatively identified as TFs associated with NAC, MYB, bHLH, ERF, WRKY, bZIP and MADS families (Figure S6). The NAC family was represented by five DEGs that were upregulated in the Jonagold apple. Moreover, five DEGs, belonging to the MYB family, were obtained and exhibited higher expression levels in the Jonagold apple than in the Granny Smith apple. In addition, some DEGs encoding ERF, WRKY and bZIP family TFs also showed higher expression levels in the Jonagold apple compared to the Granny Smith apple (Figure S6).

### 2.7. Validation of Gene Expression Levels Using qRT-PCR

**Figure 6:**
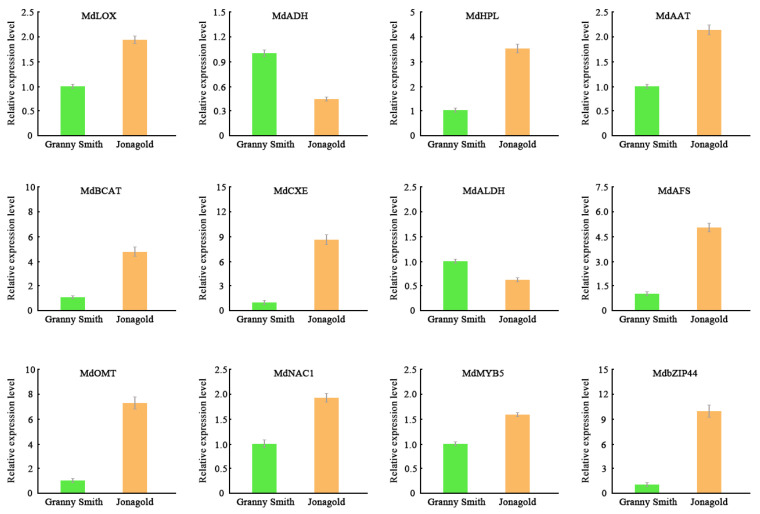
Expression profiles of selected 12 DEGs determined by qRT-PCR.

To validate the expression patterns of DEGs observed in RNA-seq datasets, 12 key genes were selected for analysis of their expression levels using qRT-PCR. These DEGs included *LOX*, *HPL*, *ADH*, *AAT*, *BCAT*, *ALDH* and *CXE* involved in fatty acid and isoleucine metabolism, *AFS* and *OMT* related to sesquiterpene and phenylpropanoid metabolism, transcription factors NAC1, MYB5 and bZIP44 that could regulate the production of volatile compounds. Except for *ADH* and *ALDH*, these DEGs all showed higher expression levels in the Jonagold apple than those in the Granny Smith apple, which were consistent with the RNA-Seq data (Figure 6). These results confirmed that the transcriptomic data in our study were accurate and reliable.

## 3. Discussion

Aroma is an important quality indicator of fruit flavor that can significantly influence the consumer choices [29]. Many factors can affect the aroma composition of apple fruit, among which cultivar is the most important [6]. In this study, volatile compounds of two famous apple varieties ‘Granny Smith’ and ‘Jonagold’ were determined using HS–SPME with GC–MS. The types of volatile compounds in the Jonagold apple were more than that in the Granny Smith apple. Among these volatiles, esters were the predominant compounds in the Jonagold apple, accounting for more than 60% of the total volatiles, which was consistent with previous report that esters were the most abundant group that contribute mainly to the sweet and fruity odors of apple fruits [11,30]. Moreover, hexyl acetate, confers a sweet and fruity odor with floral notes, was the most plentiful ester in the Jonagold apple, which was in accordance with the results of previous studies [31]. The Granny Smith apple is known to have a very low volatile emission [1]. In the present study, despite the fact that the total content of volatile compounds in the Granny Smith apple was significantly lower than that in the Jonagold apple, Granny Smith possessed more types of alcohols and aldehyde, occupied over 90% of the total volatiles. For the Granny Smith apple, 1-hexanol was the most abundant compound among alcohols and (*E*)-2-hexenal was the major aldehyde. Additionally, a range of volatiles such as α-farnesene and estragole have been detected in the Granny Smith apple, but accumulated more in Jonagold apples. The aroma volatiles of various apple varieties were different in class and quantity, and gave a characteristic sensorial perception for each type of apple fruit [32]. Previous studies have classified Jonagold into the ‘ester type’ apple based on its high ester production [5,33]. According to this classification, the Granny Smith studied here would be the ‘aldehyde type’ apple because the aldehydes in its fruit represent more than 85% of the total aromatic components. The differences in composition and concentration of volatile compounds between Granny Smith and Jonagold apples may determine diverse aroma profiles of the fruits.

In order to reveal the molecular mechanisms underlying the synthesis of volatile compounds, transcriptome sequencing of the Granny Smith and Jonagold apples was performed using the Illumina Novaseq 6000. It has been reported that at least four pathways are responsible for the production of volatile compounds in apples [6]: Straight-chain aldehydes, alcohols and esters are synthesized from fatty acid metabolism pathway [13]; Branched-chain aldehydes, alcohols and esters are derived from isoleucine pathway [34]; Sesquiterpenes are synthesized via the mevalonate pathway and phenylpropenes are synthesized from the phenylpropanoid pathway [12,35]. In this study, a total of 94 DEGs were detected in these four pathways, indicating the dramatical differences in volatile synthesis between the Granny Smith and Jonagold apples. For the formation of aldehydes, alcohols and esters in apples, β-oxidation and the lipoxygenase pathway are the two essential enzymatic systems in the catabolism of fatty acids [36,37]. The β-oxidation of long-chain fatty acids produces shorter acids such as acetic, butanoic and hexanoic acids, which can be reduced to their corresponding alcohols before being esterified with acyl-CoA by the AAT enzyme [9]. The lipoxygenase pathway contains four vital enzymes, inclusive of LOX, HPL, ADH and AAT [36]. Lipoxygenase (LOX) is a dioxygenase that catalyzes the oxygenation of polyunsaturated fatty acids. In ‘Royal Gala’ and ‘Prima’ apples, the genes *MdLOX1a* and *MdLOX5e* were found to be involved in the production of volatiles [38]. In our study, the twelve *MdLOX* genes showed significantly higher expression levels in the Jonagold apple than that in the Granny Smith apple. Combined with the measurement results of volatile profiles, the upregulated expression pattern of *MdLOX* genes in the Jonagold apple may play a key role in the volatile compounds accumulation. Products of the LOX reaction can be converted to aldehydes by the hydroperoxide lyase (HPL) enzyme and aldehydes are subsequently reduced to the corresponding alcohol by the alcohol dehydrogenase (ADH) [12]. The alcohols resulting from ADH enzymatic activity are natural substrates for the alcohol acyltransferase (AAT), which transfers an acyl group through an oxygen-dependent reaction from acyl-CoA to the OH group of an alcohol forming an ester [39]. In our study, compared to the Granny Smith apple, the *MdAAT* gene exhibited significantly higher expression levels in the Jonagold apple, accounting for the high ester production. Amino acids are the second most important source of volatile compounds in the aroma of apples. A previous study has reported that the branched amino acids, especially isoleucine, can produce the branched-chain esters in apples [34]. In our dataset, the key gene *MdBCAT*, which catalyzes the last step in the synthesis of branched amino acids, was higher expressed in the Jonagold apple than that in the Granny Smith apple, and might provide more precursors for the synthesis of branched-chain aldehydes, alcohols, acids and esters. Sesquiterpenes, which synthesized via the mevalonate pathway, are the main aroma volatiles from the isoprenoid family in the apple fruits [9]. In our study, there was only one terpenoid compound α-farnesene (sesquiterpene) detected in Granny Smith and Jonagold apples. α-Farnesene synthase (AFS) catalyzed the final step in sesquiterpene biosynthesis in apples [40]. The *MdAFS* as well as the *MdHMGS*, *MdHMGR* and *MdFPPS* genes in this pathway were all upregulated in Jonagold apples compared with the Granny Smith apple, might be responsible for high production of α-farnesene. In addition, phenylpropenes, such as eugenol, estragole and isoestragole, also contribute to apple aroma and these compounds are derived from the phenylpropanoid pathway [17,30]. It has been reported that estragole imparts a spicy and aniseed note to ‘Ellison Orange’ and ‘Royal Gala’ apples [17]. In this study, the compound estragole was detected in both two apple varieties, the key genes *MdPAL* and *MdOMT* exhibited high expression levels in Jonagold apples, possibly accelerating the reaction of enzymes in estragole biosynthesis and resulting in the accumulation of estragole.

Transcriptional regulation mediated by transcription factors (TFs) is a pivotal control hierarchy for various plant biological processes, including the synthesis of volatile compounds [41,42]. A previous study has reported that FaMYB9 is positively involved in volatile biosynthesis in strawberries, transient silencing of FaMYB9 delayed the fruit development, resulting in a significant decrease in the contents of C6 volatiles [43]. Moreover, in banana, MabZIP4 and MabZIP5 were capable of binding directly to *BanAAT* promoter, suggesting the function of MabZIPs in controlling aroma production [42]. Additionally, the transcription factor PpNAC1 was proved to transcriptionally activate the *PpAAT1* gene and formation of volatile esters in peaches [44]. AP2/ERF transcription factors have been shown to be associated with the production of volatile terpenes in sweet orange fruits [45]. In order to clarify potential transcription factors that may be involved in regulating volatile biosynthesis, expression patterns of TFs such as NAC, MYB, bZIP and ERF were analyzed. In the Jonagold apple, the MdNAC1, MdMYB5, MdERF8 and MdbZIP44 TFs had higher expression levels than in the Granny Smith apple. Combined with the aroma profiles of these two apple varieties, we speculate that these TFs can bind to the promoters of some key genes and may play important roles in regulating the synthesis of volatile compounds. Therefore, further functional studies will focus on clarifying the regulatory roles of these transcription factors in the biosynthesis of volatiles in apple.

## 4. Materials and Methods

### 4.1. Plant Materials and Sample Collection

‘Granny Smith’ and ‘Jonagold’ apple fruit samples were collected at the Baishui Apple Experimental Station of Northwest Agriculture and Forestry University in Shaanxi, China (35°21′ N, 109°55′ E). The two apple varieties were grafted onto M26 rootstock (*Malus domestica*) and planted at a density of 4 × 2 m. Unbagged apple fruits were harvested at the full ripening stage in 2020. Three biological replicates for each apple variety were prepared, with each biological replicate consisting of 8–10 fruits. The pulps of two apple varieties were carefully separated with the peels, pooled, immediately frozen in liquid nitrogen and then stored at −80 °C until use.

### 4.2. Determination of Fruit Physiological Characteristics

Measurements of apple fruit height and diameter were taken using a digital vernier caliper (Meinaite, Chengdu, China). Determinations of single fruit weight and skin color were performed by an electronic balance (MettlerToledo Inc., Greifensee, Switzerland) and a colorimeter (CR-400, Minolta, Japan), respectively. Test of apple flesh texture was conducted using a texture analyzer (FTA GS-15, Berlin, Germany; test depth 8 mm). Total soluble solid (TSS) and titratable acidity (TA) of apple fruits were measured with a hand refractometer (Atago, Tokyo, Japan) and a digital fruit acidity meter (GMK-835F Perfect, Berlin, Germany), respectively. The internal ethylene of the apples was evaluated according to the method described by Cai et al. [46] using a gas chromatograph (Agilent Technologies 7890A, Palo Alto, CA, USA) equipped with flame ionization detector and a HP-AL/S column. Measurement of CO_2_ production was performed by the method described by Contreras and Beaudry [47].

### 4.3. Volatile Compounds Extraction and GC-MS Analysis

HS-SPME was used for the extraction of volatile compounds in apples. The pulps of apple fruits were ground into powder in liquid nitrogen. For each extraction, 5 g of powder was placed into a 50 mL screwcap vial containing 1.0 g of NaCl and a magnetic stirring rotor to facilitate the release of volatiles. Prior to sealing of the vials, 10 µL of 0.4 mg/mL 3-nonanone was added as internal standard. After the headspace vial equilibrated at 50 °C for 10 min on a metal heating agitation platform, the SPME fiber coated with 50/30 µm thickness of divinylbenzene/carboxen/polydimethylsiloxane (DVB/CAR/PDMS, Supelco, Bellefonte, PA, USA) was inserted into the headspace with continuous heating and agitation (200 rpm) for 30 min to adsorb volatile compounds. Then, the fiber was introduced into the heated injector port of the chromatograph for desorption at 250 °C for 2.5 min.

The volatile compounds were analyzed with a Thermo Trace GC Ultra gas chromatograph (Thermo Fisher Scientific, Waltham, MA, USA), equipped with a 60 m × 0.25 mm × 0.25 µm HP-INNOWax capillary column. Helium was circulated as the carrier gas with a flow of 1.0 mL/min in a splitless mode. The initial oven temperature was kept at 40 °C for 3 min and increased to 150 °C at a rate of 5 °C/min, then increased at 10 °C/min to 220 °C and held for 5 min. The temperature of ion source and transfer line were both 240 °C. Mass spectra were operated in electron ionization mode of 70 eV with the scan range from m/z 35 to 450.

Identification of volatile compounds was based on mass spectra matching with the database of NIST/EPA/NIH Mass Spectral Library (NIST 2014), the retention times with an in-house developed retention time library based on commercial standards, and the retention indices with those of authentic compounds or literature data, which calculated under the same chromatographic conditions after the injection of a C7-C30 n-alkane series (Supelco, Bellefonte, PA, USA). Quantification was carried out by the internal standard method, as illustrated by Qin et al. [48], where the concentration of each volatile compound was normalized to that of 3-nonanone.

### 4.4. RNA Extraction, Library Preparation and Transcriptome Sequencing

Total RNA was isolated from the pulp of the two apple varieties (180 days after flowering) using the RNAprep pure Plant Kit (Tiangen, Beijing, China) according to the manufacturer’s instructions. Quality and quantity of the total RNA were assessed using a Qubit 2.0 fluorometer RNA Assay Kit (Invitrogen Inc., Carlsbad, CA, USA) and Agilent 2100 Bioanalyzer (Agilent, Palo Alto, CA, USA). A total amount of 3 µg of the high-quality RNA per sample was used for the cDNA library preparation and 6 libraries (3 biological replicates) were constructed by NEBNext^®^ UltraTM RNA Library Prep Kit for Illumina^®^ (NEB, Ipswich, MA, USA). Transcriptome sequencing was performed using Illumina Novaseq platform (Illumina, San Diego, CA, USA) to paired-end reads by Novogene (Beijing, China).

Quantitative real-time reverse transcription PCR (qRT-PCR) was performed with an iQ5 Multicolor Real-Time PCR Detection System (Bio-Rad, Hercules, CA, USA) using SYBR^®^ Green Master Mix (TaKaRa, Kyoto, Japan). Each sample was assessed in three biological repeats and normalized using apple *MdActin* and *MdEF-1α* genes as the internal control. The relative expression of the genes was analyzed by the 2^−ΔΔCT^ method and the primers used for qRT-PCR were presented in Table S1.

### 4.5. Differential Expression Analysis and Functional Annotations

In order to obtain the clean reads, the raw reads were filtered by removing the adaptor, low-quality reads (Qphred ≤ 20 bases) and unknown sequences. Then, clean reads were mapped to apple reference genome (https://iris.angers.inra.fr/gddh13, accessed on 1 July 2017). Quantification of the gene expression levels was estimated as fragments per kilobase of transcript per million mapped reads (FPKM). Cufflinks (version 2.2.1) software was used in the process of quantifying gene expression, and the counts were then normalized to the FPKM values. DESeq R package (version 1.18.0) was used to identify the differentially expressed genes (DEGs) between the two apple varieties. A fold change (FC) value of ≥2 and a false discovery rate (FDR) of <0.05 were used as criteria for selecting DEGs. GOseq R package was used to analyze Gene Ontology (GO) enrichment. GO terms with corrected *p*-values < 0.05 were assigned as significantly enriched by DEGs. Kyoto Encyclopedia of Genes and Genomes (KEGG) enrichment analysis of DEGs was conducted using the KOBAS software (version 3.0).

### 4.6. Statistical Analysis

Data were presented as mean values of three biological replicates ± standard error (SE). Statistical analysis and one-way analysis of variance (ANOVA) were performed by SPSS v19.0 software (SPSS Ins., Chicago, IL, USA), and significant differences between the samples were assessed by the Duncan test (*p* < 0.05).

## 5. Conclusions

In this study, we investigated the volatile profiles of the Granny Smith and Jonagold apples and found that aldehydes were the most abundant types of volatile compounds contributing to the apple aroma in Granny Smith while esters were the main types of volatile compounds in the Jonagold apple. The analysis of transcriptome sequencing revealed 94 DEGs in the pathways of fatty acid metabolism, amino acid metabolism, mevalonate and phenylpropanoid. Compared with the Granny Smith apple, the key genes such as *MdLOX*, *MdAAT*, *MdAFS*, *MdPAL* and *MdOMT*, and the important transcription factors such as MdNAC, MdMYB, MdERF and MdbZIP all showed higher expression levels in the Jonagold apple, resulting in the accumulation of some esters as well as the α-farnesene and estragole, and might be responsible for the high production of volatiles. Furthermore, qRT-PCR experiments verified that those genes were differentially expressed between the two apple varieties. Production of aroma in apple fruits was the result of a combination of complex metabolic pathways, including the diverse physiological processes and molecular regulation mechanisms. Our study provides valuable clues to gain better insight into the aroma formation process of fruits.

## Figures and Tables

**Table 1:** Physiological traits of Granny Smith and Jonagold apples.

Trait	Granny Smith	Jonagold
Fresh weight (g)	205 ± 15	240 ± 18
Height (mm)	50.05 ± 1.23	56.20 ± 1.85
Diameter (mm)	57.80 ± 2.10	61.55 ± 2.35
L* value	63.52 ± 0.65	58.62 ± 1.67
a* value	−17.62 ± 1.85	13.38 ± 2.30
b* value	37.95 ± 1.52	29.13 ± 2.58
TSS (°Brix)	13.7 ± 0.2	14.2 ± 0.2
TA (%)	0.40 ± 0.04	0.32 ± 0.03
Firmness (kg/cm^2^)	7.05 ± 0.15	6.58 ± 0.12
Internal ethylene (μL/L)	5 ± 1	62 ± 8
CO_2_ production (μmol/kg·s)	70 ± 8	48 ± 5

Values are means ± standard deviation; TSS—total soluble solid; TA—titratable acidity.

**Table 2:** The contents (µg/kg) of volatile compounds detected in Granny Smith and Jonagold apples.

Class	Compounds	CAS No	RT	RI	Granny Smith	Jonagold
Ester	Propyl acetate	109-60-4	10.67	982	–	7.99 ± 0.21
	Isobutyl acetate	110-19-0	11.72	1020	–	2.40 ± 0.11
	Propyl propionate	106-36-5	12.57	1050	–	1.40 ± 0.02
	Butyl acetate	123-86-4	13.40	1074	-	213.44 ± 15.74
	2-Methylbutyl acetate	624-41-9	14.84	1126	–	85.03 ± 6.17
	Isobutyl butanoate	539-90-2	15.29	1155	–	1.19 ± 0.03
	Butyl propionate	590-01-2	15.41	1157	–	8.87 ± 0.24
	Amyl acetate	628-63-7	16.37	1178	–	14.65 ± 1.35
	Butyl butanoate	109-21-7	17.69	1240	–	5.75 ± 0.17
	Butyl 2-methylbutanoate	15706-73-7	18.08	1243	–	19.21 ± 1.08
	Hexyl acetate	142-92-7	19.28	1274	1.25 ± 0.06	885.38 ± 25.44
	(*E*)-2-Hexenyl acetate	2497-18-9	21.01	1338	–	1.84 ± 0.06
	Hexyl propanoate	2445-76-3	21.14	1347	–	8.40 ± 0.54
	Heptyl acetate	112-06-1	22.09	1386	–	3.16 ± 0.26
	Butyl hexanoate	626-82-4	23.17	1410	–	1.75 ± 0.10
	Hexyl butanoate	2639-63-6	23.23	1423	0.35 ± 0.02	27.19 ± 1.58
	Hexyl 2-methylbutyrate	10032-15-2	23.53	1438	0.46 ± 0.03	31.95 ± 2.13
	Heptyl formate	112-23-2	24.15	1455	–	1.59 ± 0.08
	Octyl acetate	112-14-1	24.81	1483	–	5.56 ± 0.42
	Hexyl hexanoate	6378-65-0	28.07	1593	–	6.60 ± 0.38
Alcohol	1-Butanol	71-36-3	15.35	1156	–	19.42 ± 1.02
	2-Methyl-1-butanol	137-32-6	17.20	1210	0.91 ± 0.05	6.42 ± 0.38
	2-Hexyn-1-ol	764-60-3	17.41	1225	0.85 ± 0.05	–
	1-Hexanol	111-27-3	21.40	1361	1.00 ± 0.04	74.69 ± 4.81
Aldehyde	Hexanal	66-25-1	13.76	1090	7.95 ± 0.58	23.93 ± 1.84
	(*Z*)-3-Hexenal	6789-80-6	15.60	1161	3.32 ± 0.27	–
	(*E*)-2-Hexenal	6728-26-3	17.93	1240	40.24 ± 3.62	86.21 ± 6.92
	(*Z*)-2-Heptenal	57266-86-1	21.03	1339	1.42 ± 0.08	–
	1-Nonanal	124-19-6	22.79	1401	0.50 ± 0.03	1.66 ± 0.20
	(*E*)-2-Octenal	2548-87-0	23.89	1443	1.90 ± 0.10	2.12 ± 0.06
	(*Z*)-2-Nonenal	60784-31-8	26.63	1531	0.81 ± 0.06	3.47 ± 0.12
	(*E*)-2-Decenal	3913-81-3	29.08	1655	–	3.88 ± 0.23
Ketone	1-Octen-3-one	4312-99-6	20.22	1305	0.73 ± 0.05	–
phenylpropene	Estragole	140-67-0	29.66	1687	1.04 ± 0.10	530.16 ± 47.66
Terpenoid	α-Farnesene	502-61-4	30.75	1754	1.65 ± 0.11	31.15 ± 2.85

Values are means ± standard deviation; CAS No: CAS number; RT: retention time; RI: retention index; –: indicates not detected.

## Data Availability

The transcriptomic data generated in this study are submitted to NCBI Gene Expression Omnibus repository (http://www.ncbi.nlm.nih.gov/geo/, accessed on 7 March 2022) under accession number GSE176136.

## Supplementary Materials

The following supporting information can be downloaded at: https://www.mdpi.com/article/10.3390/ijms23062939/s1.
