# Supplementary material for: GC-MS Metabolite and Transcriptome Analyses Reveal the Differences of Volatile Synthesis and Gene Expression Profiling between Two Apple Varieties
Source: Int J Mol Sci. 2022 Mar 9;23(6):2939. doi: 10.3390/ijms23062939 (PMC8951106; doi:10.3390/ijms23062939)
Supplement: Supplementary file 1 [file ijms-23-02939-s001.zip › ijms-1618744-supplementary.pdf]

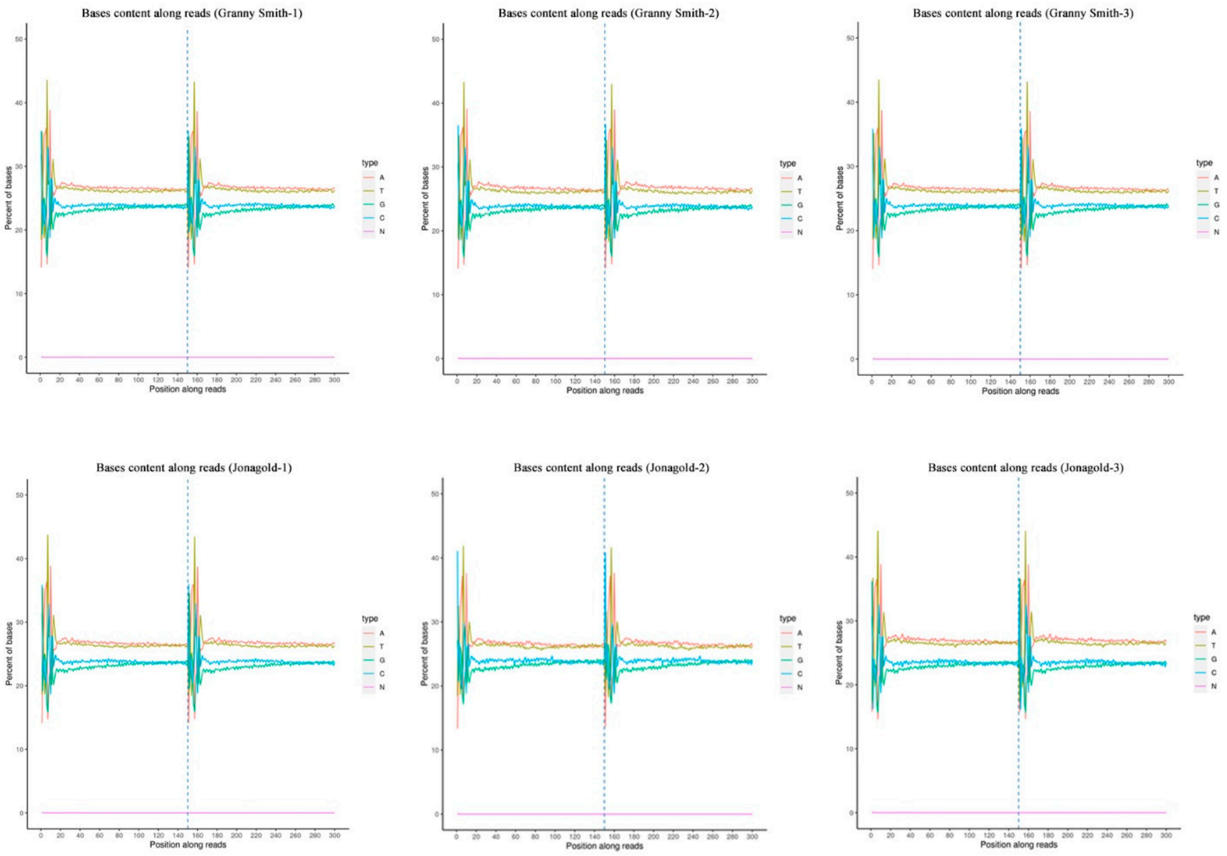

Figure S1. Base contents of reads in Granny Smith and Jonagold apples.

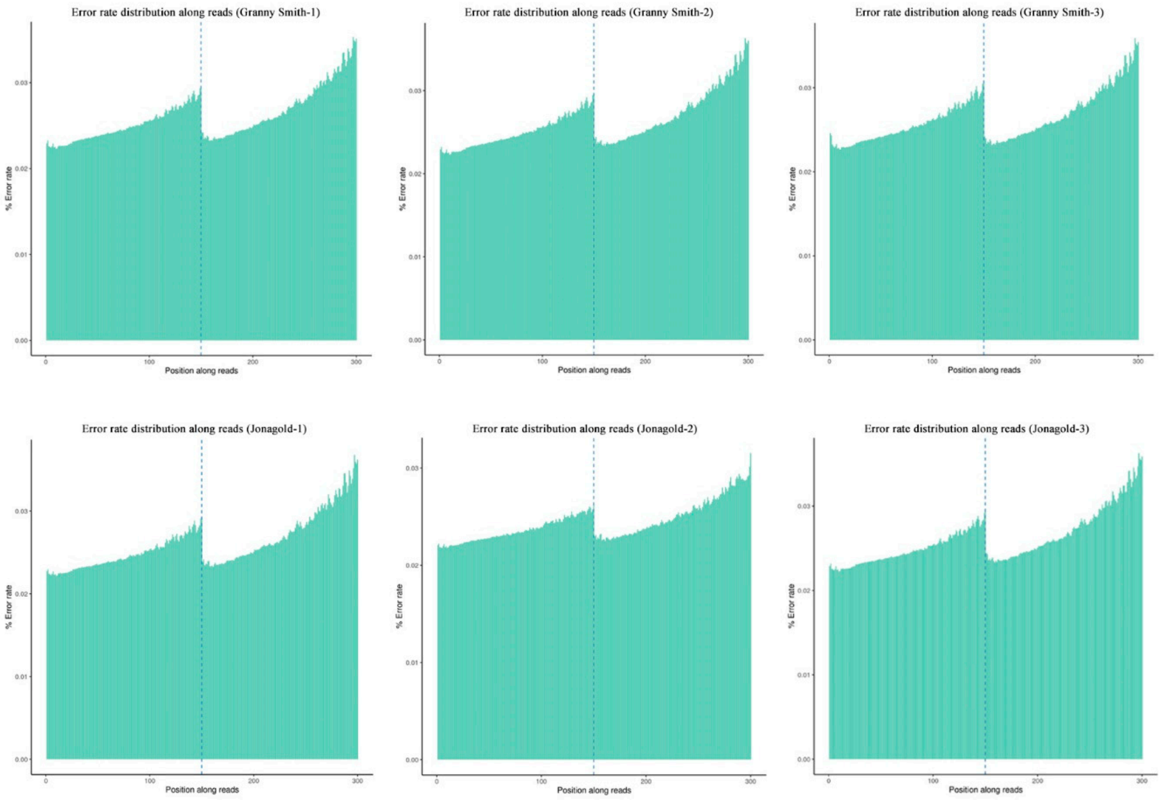

Figure S2. Error rate distribution among reads in Granny Smith and Jonagold apples.

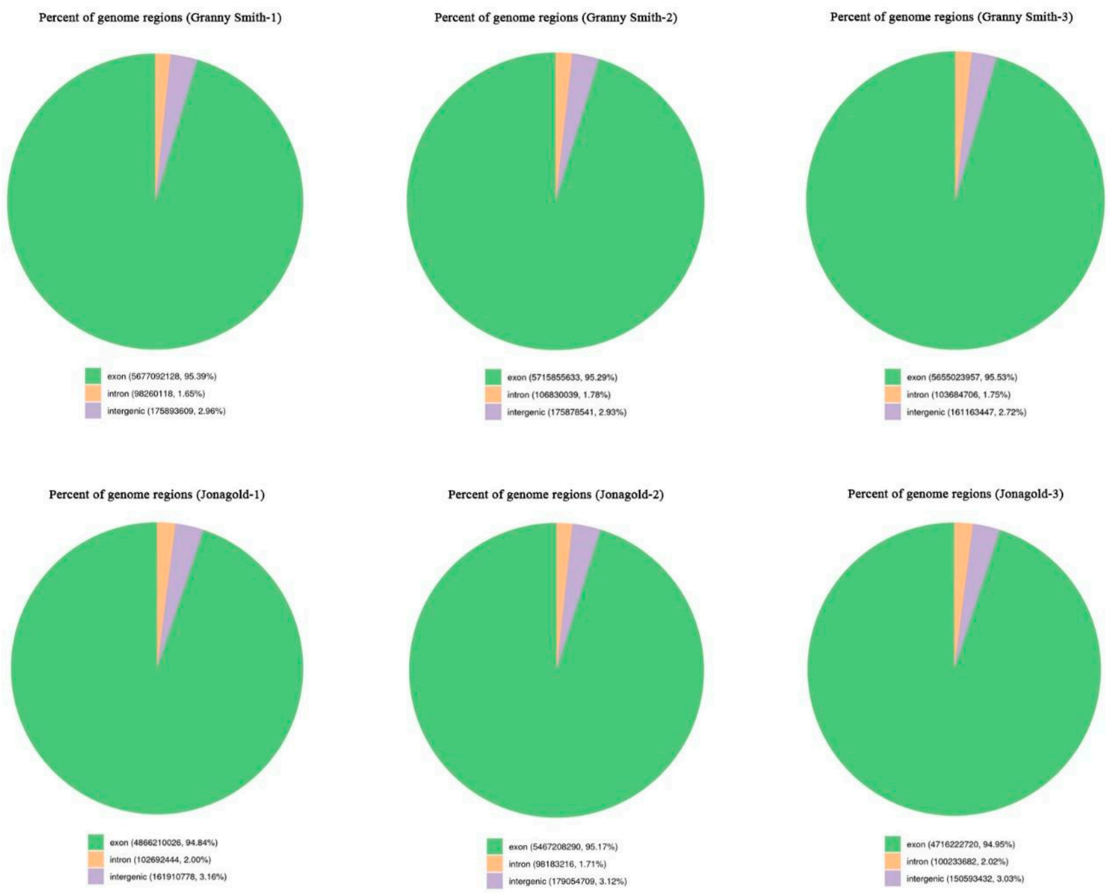

Figure S3. Percent of reads mapped to genome regions in Granny Smith and Jonagold apples.

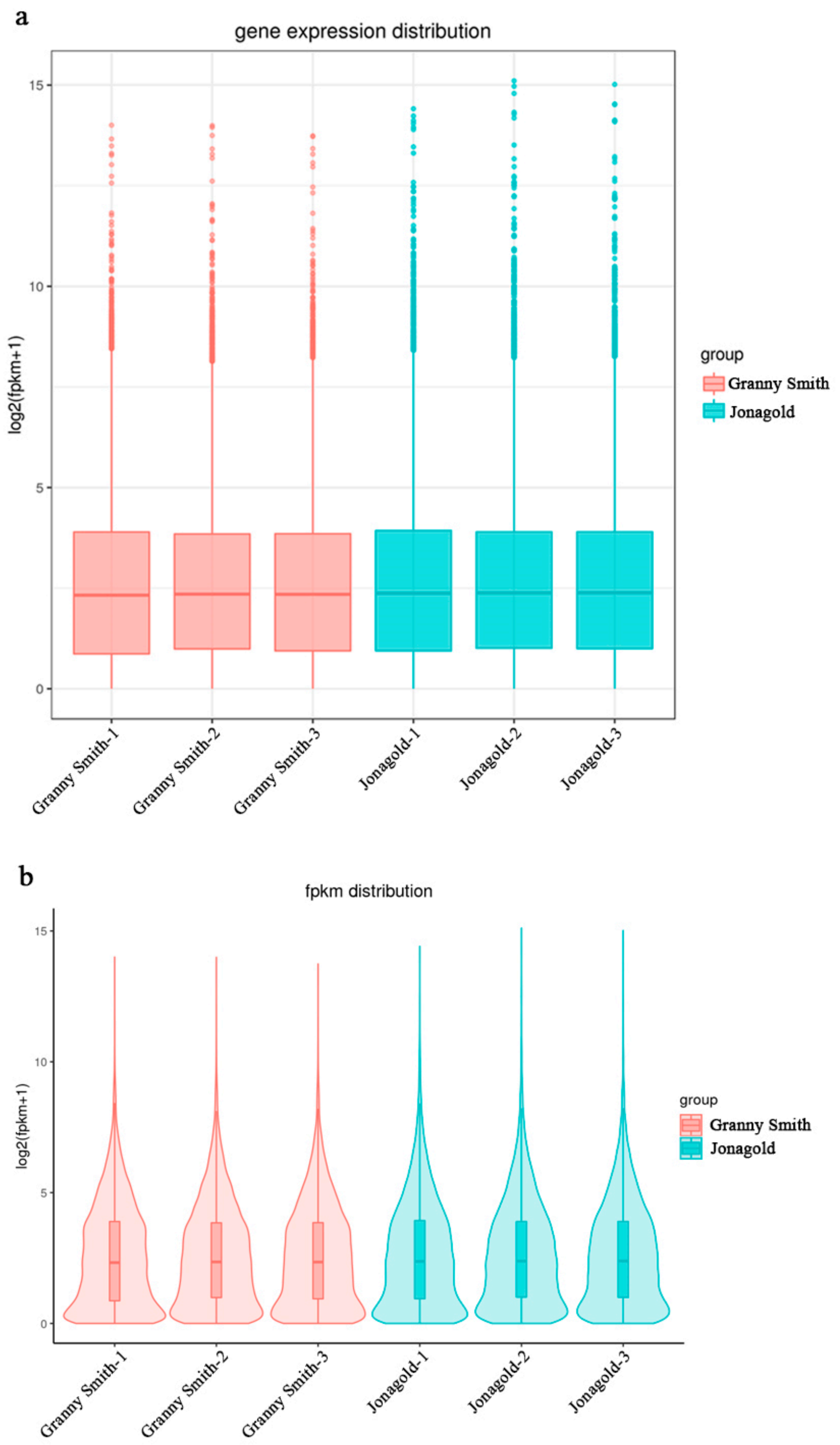

Figure S4. Distribution of high-throughput sequencing results in Granny Smith and Jonagold apples, (**a**) gene expression distribution in Granny Smith and Jonagold apples, and (**b**) fpkm distribution in Granny Smith and Jonagold apples.

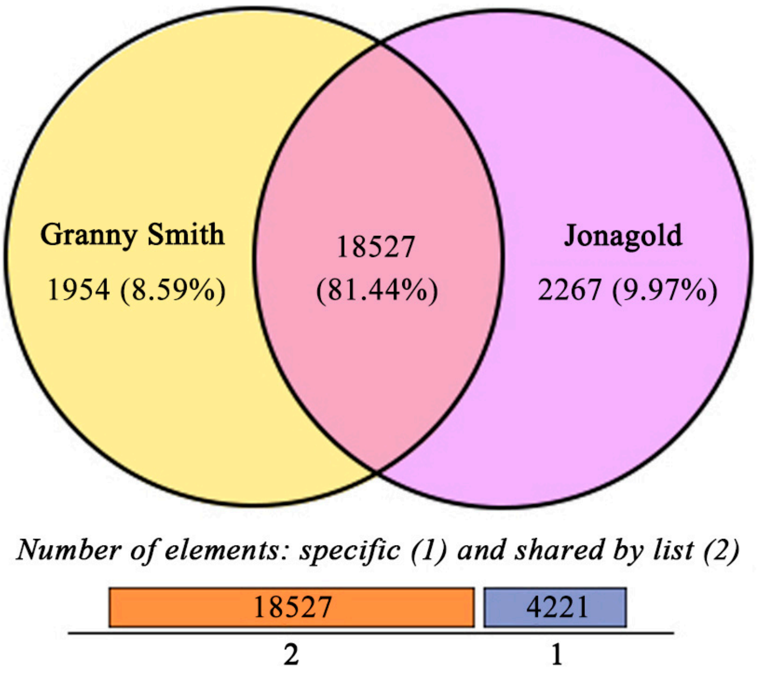

Figure S5. Numbers of unique genes and shared genes between Granny Smith and Jonagold apples.

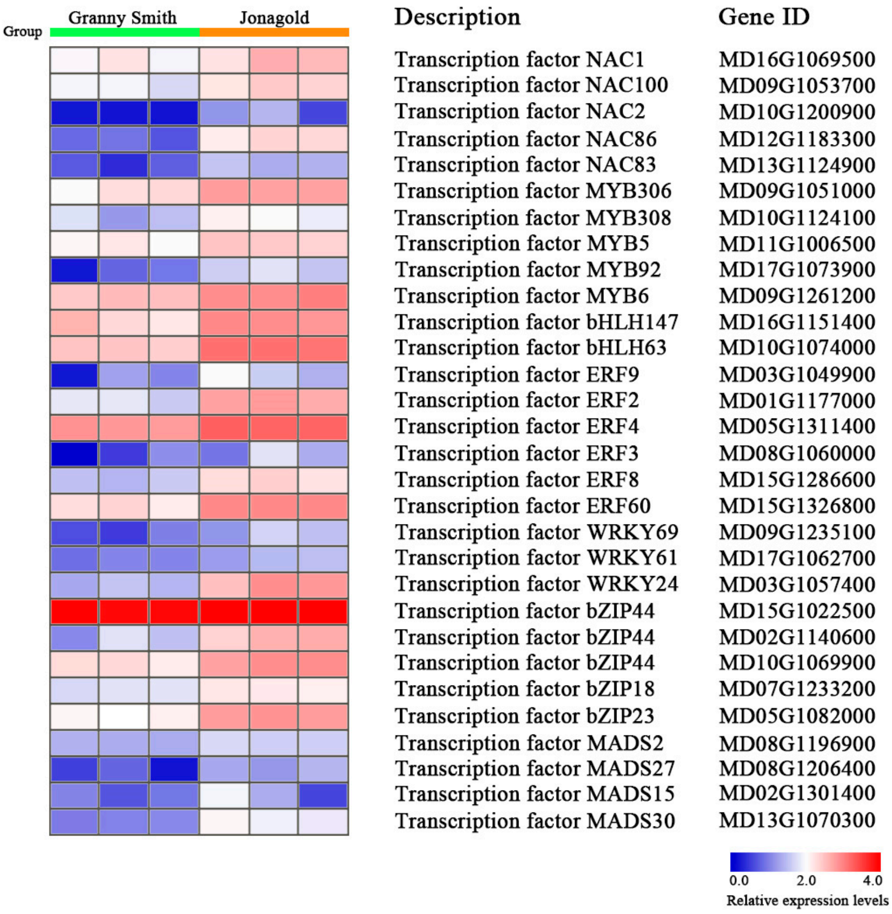

Figure S6. Analysis of transcription factors (TFs) associated with volatile compounds in Granny Smith and Jonagold apples.

Table S1. Twelve pairs of primers used for qRT-PCR in Granny Smith and Jonagold apples.

Gene ID	Gene Name	Forward Primer (5′–3′)	Reverse Primer (3′–5′)
MD05G1156900	*MdLOX-8*	ACCTGTCTTGGGTGGG	ATTGTTTCTTGGGGCT
MD05G1179800	*MdADH-5*	TAATCACTTCGTCGGC	CCTTTTTCGGTTTTGC
MD08G1038600	*MdHPL*	ACGGCGGAAAAATCAC	GAACCCGAACAGCAAC
MD02G1015200	*MdAAT-1*	ATTGTCCTTTGCTGCT	TTCTTGGTGGATTTCG
MD15G1063100	*MdBCAT-1*	CCACATCGGGGACTAT	GAGCAACACCAACAGC
MD02G1198400	*MdCXE-7*	ACGACACCTGTTCCGA	GACCATAGTTCCCCCG
MD13G1090800	*MdALDH-1*	TGCATCAGGGAAAACT	AAAGCGGAAAAGGACT
MD10G1311000	*MdAFS*	GCATTATTGGTGGAGG	AGGCGAACGATGAGAG
MD05G1308800	*MdOMT-3*	GCTTGTCTCCGTTCGT	TAGCCATTGCCTTGTT
MD16G1069500	MdNAC1	CAAATACCAAGCCACC	TTCCAATAACCCGACA
MD11G1006500	MdMYB5	GGTTTGAAGAGAGGGC	AGGGGCGGAGATAGTT
MD02G1140600	MdbZIP44	ACTCCACCCTCCTTCG	CTCCTGTTTTCGCATC

Table S2. Total content (μg/kg) of each type of volatiles in Granny Smith and Jonagold apples.

Class	Granny Smith	Jonagold
Ester	2.06 ± 0.06	1333.35 ± 38.14
Alcohol	2.76 ± 0.08	100.53 ± 5.02
Aldehyde	56.14 ± 4.13	121.27 ± 7.56
Ketone	0.73 ± 0.05	–
Phenylpropene	1.04 ± 0.10	530.16 ± 47.66
Terpenoid	1.65 ± 0.11	31.15 ± 2.85

Datas are the mean value ± standard deviation (n = 3); –: indicates not detected.

Table S3. Summary statistics of sequencing data assembly and quality in Granny Smith and Jonagold apples.

Sample	Raw Reads	Clean Reads	Clean Bases	Q20	Q30	GC Content
Granny Smith-1	4468230	43696070	6.55G	97.59%	93.37%	47.15%
Granny Smith-2	44667654	43670016	6.55G	97.52%	93.22%	47.12%
Granny Smith-3	43889832	42893502	6.43G	97.51%	93.01%	47.24%
Jonagold-1	41877640	41011642	6.15G	97.60%	93.33%	46.95%
Jonagold-2	47117690	46290824	6.94G	98.33%	94.78%	47.23%
Jonagold-3	43442248	42419868	6.36G	97.60%	93.30%	46.48%

Table S4. Statistics of the clean reads mapped to apple reference genome for each sample.

Sample	Positive Map	Negative Map	Total Map
Granny Smith-1	19151707 (43.83%)	19185476 (43.91%)	39799279 (91.08%)
Granny Smith-2	19237468 (44.05%)	19279996 (44.15%)	40116235 (91.86%)
Granny Smith-3	19059252 (44.43%)	19103030 (44.54%)	39591068 (92.30%)
Jonagold-1	16632663 (40.56%)	16682529 (40.68%)	34295995 (83.63%)
Jonagold-2	18566697 (40.11%)	18613306 (40.21%)	38400479 (82.95%)
Jonagold-3	16061050 (37.86%)	16108069 (37.97%)	33209471 (78.29%)

Table S5. Differentially expressed genes involved in fatty acid and isoleucine metabolism pathways between Granny Smith and Jonagold apples.

Pathway	Gene ID	Gene Name	FPKM in Granny Smith-1	FPKM in Granny Smith-2	FPKM in Granny Smith-3	FPKM in Jonagold-1	FPKM in Jonagold-2	FPKM in Jonagold-3	Annotation
Fatty acid metabolism	MD16G1238600	*MdACC-1*	1082.16	1213.45	1072.24	5261.40	4484.58	4896.25	Acetyl-CoA carboxylase
	MD01G1022300	*MdACC-2*	486.84	363.38	460.91	3091.67	2415.87	2399.53	Acetyl-CoA carboxylase
	MD13G1233600	*MdACC-3*	871.20	790.46	901.71	6499.30	4207.73	4404.13	Acetyl-CoA carboxylase
	MD11G1056800	*MdMAT-1*	1004.44	885.18	821.27	4087.67	3537.57	3333.93	Acyl-carrier-protein
	MD03G1055800	*MdMAT-2*	345.92	245.79	361.17	1520.54	1201.89	1296.94	Acyl-carrier-protein
	MD08G1135900	*MdKASIII-1*	669.63	617.34	603.28	1535.35	1440.30	1351.76	3-Oxoacyl-synthase III
	MD09G1022600	*MdKASIII-2*	35.02	12.25	27.35	254.25	173.58	169.44	3-Oxoacyl-synthase III
	MD14G1002100	*MdKAR-1*	355.31	271.92	243.73	1107.08	803.09	679.00	3-Oxoacyl-reductase
	MD13G1112000	*MdKAR-2*	229.76	196.80	223.62	5731.63	4461.51	4174.89	3-Oxoacyl-reductase
	MD16G1111600	*MdKAR-3*	830.20	512.82	616.15	6881.90	5126.18	5321.09	3-Oxoacyl-reductase
	MD13G1166700	*MdDH*	1416.12	902.33	1069.82	7101.59	3779.26	4344.33	3-Hydroxyacyl-CoA dehydrogenase
	MD12G1059500	*MdER-1*	544.92	328.27	415.86	7265.74	4563.68	4795.33	Enoyl reductase
	MD14G1058600	*MdER-2*	582.51	299.69	404.60	3257.06	2095.07	2205.18	Enoyl reductase
	MD10G1264200	*MdKASI-1*	32.46	17.15	28.96	354.22	479.00	397.43	3-Oxoacyl-synthase I
	MD14G1131500	*MdKASI-2*	4.27	0.82	1.61	135.76	86.79	68.52	3-Oxoacyl-synthase I
	MD05G1286700	*MdKASI-3*	949.77	850.07	900.90	2419.03	1772.08	1734.24	3-Oxoacyl-synthase I
	MD15G1221800	*MdKAS II*	2139.56	1910.81	2310.98	1287.27	671.26	727.58	3-Oxoacyl-synthase II
	MD02G1127500	*MdOTE-1*	21.35	29.40	12.87	914.54	705.32	758.73	Oleate-ACP thioesterase
	MD05G1172000	*MdOTE-2*	234.88	164.13	189.83	549.22	533.93	517.03	Oleate-ACP thioesterase
	MD15G1152700	*MdFAD*	88.83	230.28	156.05	11.11	34.06	16.20	Acyl-lipid omega-6 desaturase
	MD07G1004500	*MdLOX-1*	14723.22	11271.34	7755.02	84124.37	74374.61	76101.12	Lipoxygenase
	MD07G1004600	*MdLOX-2*	5245.11	3166.72	2853.94	31906.56	22469.05	26600.45	Lipoxygenase
	MD07G1003600	*MdLOX-3*	1959.34	1935.31	1041.67	5525.52	4161.59	5181.55	Lipoxygenase
	MD02G1317800	*MdLOX-4*	724.29	868.85	1130.96	1068.82	3741.91	1951.02	Lipoxygenase
	MD17G1167100	*MdLOX-5*	0.85	1.63	1.61	9.87	16.48	3.74	Lipoxygenase
	MD05G1157000	*MdLOX-6*	199.86	199.25	159.27	494.91	410.89	454.74	Lipoxygenase
	MD11G1103700	*MdLOX-7*	36.73	18.78	22.52	48.13	65.92	53.57	Lipoxygenase
	MD05G1156900	*MdLOX-8*	13.67	17.15	12.07	30.86	32.96	42.36	Lipoxygenase
	MD13G1113800	*MdLOX-9*	10.25	7.35	15.28	45.67	25.27	31.15	Lipoxygenase
	MD09G1069500	*MdLOX-10*	2857.01	2685.75	1384.34	88900.73	80688.40	89279.88	Lipoxygenase
	MD04G1204000	*MdLOX-11*	2.56	9.80	5.63	1249.01	424.07	534.48	Lipoxygenase
	MD12G1218200	*MdLOX-12*	16.23	12.25	16.09	46.90	52.73	42.36	Lipoxygenase

	MD08G1038600	*MdHPL*	5.98	26.13	20.11	59.24	62.62	54.82	Hydroperoxide lyase
	MD10G1014500	*MdADH-1*	376.66	354.40	397.36	2.47	2.20	1.25	Alcohol dehydrogenase
	MD13G1165800	*MdADH-2*	59.79	46.55	70.79	352.98	307.61	299.01	Alcohol dehydrogenase
	MD10G1013800	*MdADH-3*	417.66	761.87	570.30	7.41	4.39	6.23	Alcohol dehydrogenase
	MD10G1014200	*MdADH-4*	1288.86	2094.54	1443.06	280.16	125.24	179.40	Alcohol dehydrogenase
	MD05G1179800	*MdADH-5*	444.14	562.63	520.43	124.65	108.76	85.96	Alcohol dehydrogenase
	MD10G1013900	*MdADH-6*	320.29	529.15	398.97	83.93	28.56	33.64	Alcohol dehydrogenase
	MD06G1121600	*MdADH-7*	626.92	243.34	353.12	117.25	139.53	85.96	Alcohol dehydrogenase
	MD02G1015200	*MdAAT-1*	381.79	245.79	144.79	1242.84	852.53	1249.60	Alcohol acyltransferase
	MD14G1011700	*MdAAT-2*	19.64	48.18	32.98	9.87	7.69	12.46	Alcohol acyltransferase
	MD15G1386800	*MdAAT-3*	1.71	4.90	4.83	41.96	51.64	64.78	Alcohol acyltransferase
	MD10G1283900	*MdAAT-4*	25.62	17.15	22.52	96.27	64.82	27.41	Alcohol acyltransferase
	MD13G1109300	*MdAAT-5*	250.26	60.43	57.11	15181.91	10423.74	11219.01	Alcohol acyltransferase
	MD13G1109000	*MdAAT-6*	4.27	15.52	1.61	302.38	778.92	951.84	Alcohol acyltransferase
	MD16G1108900	*MdAAT-7*	67.48	77.58	44.24	202.41	247.19	210.55	Alcohol acyltransferase
	MD04G1147900	*MdAAT-8*	24.77	90.64	61.13	3029.96	6025.95	4010.44	Alcohol acyltransferase
	MD02G1175300	*MdAAT-9*	31.60	12.25	22.52	176.49	71.41	77.24	Alcohol acyltransferase
	MD02G1175400	*MdAAT-10*	31.60	5.72	8.85	80.22	72.51	64.78	Alcohol acyltransferase
Isoleucine metabolism	MD09G1187000	*MdTS-1*	304.92	327.45	314.51	59.24	49.44	38.62	Threonine synthase
	MD15G1212700	*MdTS-2*	70.89	106.97	81.24	319.66	329.59	272.84	Threonine synthase
	MD02G1085500	*MdTS-3*	231.46	293.15	234.07	2959.61	2576.27	2319.80	Threonine synthase
	MD06G1028700	*MdALS*	538.95	587.13	460.91	1318.13	1330.43	1335.57	Acetolactate synthase
	MD17G1231900	*MdAHIR*	843.86	683.48	690.16	1634.08	1957.75	2187.74	Acetohydroacid isomeroreductase
	MD04G1003600	*MdDHAD*	544.92	547.11	700.61	150.57	176.88	165.70	Dihydroxy-acid dehydratase
	MD15G1063100	*MdBCAT-1*	5.98	15.52	13.67	32.09	71.41	49.83	Branched-chain amino acid aminotransferase
	MD05G1110500	*MdBCAT-2*	11.96	9.80	7.24	92.57	62.62	97.18	Branched-chain amino acid aminotransferase
	MD12G1173200	*MdPD-1*	10813.94	9242.12	10696.63	1621.74	1498.52	1446.45	Pyruvate decarboxylase
	MD12G1172500	*MdPD-2*	9902.60	9121.26	10148.05	2775.72	2845.43	2549.04	Pyruvate decarboxylase
	MD12G1172400	*MdPD-3*	12272.76	7935.58	11752.78	3259.52	3034.40	2524.12	Pyruvate decarboxylase
	MD04G1159900	*MdPD-4*	8966.49	10483.33	4076.59	56710.30	46525.61	46227.79	Pyruvate decarboxylase
	MD13G1090800	*MdALDH-1*	149.47	160.87	115.83	22.22	15.38	9.97	Aldehyde dehydrogenase(NAD+)
	MD13G1090600	*MdALDH-2*	703.79	948.06	711.07	2697.96	1650.13	2043.22	Aldehyde dehydrogenase(NAD+)
	MD02G1050000	*MdALDH-3*	29.89	37.56	28.96	13.58	9.89	9.97	Aldehyde dehydrogenase(NAD+)
	MD00G1029100	*MdALDH-4*	40.14	81.66	34.59	155.51	246.09	190.62	Aldehyde dehydrogenase(NAD+)
	MD10G1091000	*MdCXE-1*	76.87	49.81	46.65	392.48	411.98	419.86	Carboxylesterase

MD05G1191100	*MdCXE-2*	952.34	914.58	847.01	50.60	131.83	108.39	Carboxylesterase
MD11G1176900	*MdCXE-3*	28.19	54.71	51.48	7.41	8.79	8.72	Carboxylesterase
MD15G1085200	*MdCXE-4*	678.17	627.14	604.09	87.63	131.83	93.44	Carboxylesterase
MD10G1091100	*MdCXE-5*	1.71	4.08	6.44	361.62	663.57	637.88	Carboxylesterase
MD08G1226300	*MdCXE-6*	112.74	88.19	128.70	413.46	398.80	313.96	Carboxylesterase
MD02G1198400	*MdCXE-7*	2.56	3.27	0.80	35.79	67.02	32.39	Carboxylesterase
MD02G1198500	*MdCXE-8*	266.48	345.42	228.44	440.61	793.21	661.55	Carboxylesterase

Table S6. Differentially expressed genes involved in sesquiterpene and phenylpropanoid metabolism between Granny Smith and Jonagold apples.

Pathway	Gene ID	Gene Name	FPKM in Granny Smith-1	FPKM in Granny Smith-2	FPKM in Granny Smith-3	FPKM in Jonagold-1	FPKM in Jonagold-2	FPKM in Jonagold-3	Annotation
Sesquiterpene metabolism	MD06G1070000	*MdHMGS*	76.02	35.11	33.78	407.29	294.43	300.25	Hydroxymethylglutaryl-CoA synthase
	MD15G1227900	*MdHMGR*	243.42	192.71	456.08	1569.90	1327.14	1425.27	Hydroxymethylglutaryl-CoA reductase
	MD09G1247700	*MdPMK*	146.91	181.28	170.53	59.24	62.62	52.33	Phosphomevalonate kinase
	MD02G1154400	*MdFPPS-1*	1045.44	868.85	680.50	6369.71	4547.20	3627.96	Geranylgeranyl diphosphate synthase
	MD17G1260500	*MdFPPS-2*	0.01	0.02	0.01	19.75	23.07	14.95	Geranylgeranyl diphosphate synthase
	MD10G1311000	*MdAFS*	761.02	953.77	812.42	10068.61	7828.79	10345.66	Alpha-farnesene synthase
Phenylpropanoid metabolism	MD12G1116700	*MdPAL-1*	2.56	7.35	3.22	60.48	47.24	19.93	Phenylalanine ammonia-lyase
	MD01G1106900	*MdPAL-2*	42.71	29.40	44.24	133.29	75.81	54.82	Phenylalanine ammonia-lyase
	MD03G1121400	*MdPAL-3*	5.98	5.72	0.80	11.11	21.97	24.92	Phenylalanine ammonia-lyase
	MD00G1221400	*MdC4H-1*	3619.74	2577.96	3186.15	1785.89	1153.55	1097.61	Cinnamate 4-hydroxylase
	MD03G1050900	*MdC4H-2*	1059.96	736.56	982.15	504.79	284.54	312.71	Cinnamate 4-hydroxylase
	MD03G1051100	*MdC4H-3*	41.00	27.76	45.85	3.70	6.59	2.49	Cinnamate 4-hydroxylase
	MD03G1051000	*MdC4H-4*	5.98	2.45	4.83	23.45	16.48	12.46	Cinnamate 4-hydroxylase
	MD17G1229400	*Md4CL*	70.89	111.06	149.61	32.09	15.38	4.98	4-Coumarate-CoA ligase
	MD17G1222400	*MdCCoAR*	2714.37	1904.28	2243.41	793.59	745.97	566.87	Cinnamoyl-CoA reductase
	MD01G1042500	*MdCADH-1*	0.01	0.01	0.01	13.58	6.59	9.97	Cinnamyl-alcohol dehydrogenase
	MD01G1042800	*MdCADH-2*	0.01	0.01	0.02	9.87	6.59	7.48	Cinnamyl-alcohol dehydrogenase
	MD01G1051900	*MdOMT-1*	904.51	578.14	898.49	93261.16	72243.28	83026.88	O-methyltransferase
	MD05G1308600	*MdOMT-2*	0.01	0.02	0.02	341.87	210.94	186.88	O-methyltransferase
	MD05G1308800	*MdOMT-3*	2.56	1.63	3.22	41.96	30.76	24.92	O-methyltransferase
